# Off-Target Anti-Inflammatory Activity of the P2X7 Receptor Antagonist AZ11645373

**DOI:** 10.1007/s10753-016-0499-8

**Published:** 2017-01-19

**Authors:** Olga V. Oskolkova, Viktoria Godschachner, Valery N. Bochkov

**Affiliations:** Department of Pharmaceutical Chemistry, Institute of Pharmaceutical Sciences, University of Graz, Humboldtstrasse 46/III, 8010 Graz, Austria

**Keywords:** purine X receptors, P2X7 receptor, oxidized phospholipids, anti-inflammatory activity

## Abstract

We have found that a well-characterized P2X7 receptor antagonist AZ11645373 blocked production of pro-inflammatory chemokine IL-8 in endothelial cells treated with OxPAPC. The effect was not due to toxicity of AZ11645373 as documented by cellular metabolic activity assay. The mechanism of inhibition by AZ11645373 was apparently independent of the P2X7 receptor because this receptor was not involved in induction of IL-8 under our experimental conditions. In support of this notion, two P2X7 agonists ATP and BzATP did not upregulate IL-8. On the other hand, a chemically different P2X7 receptor antagonist A740003 did not inhibit OxPAPC-induced production of IL-8. The inhibitory action of AZ11645373 was observed at the level of IL-8 protein and messenger RNA (mRNA) induction. Furthermore, AZ11645373 inhibited induction of mRNA encoding for COX-2 (*PTGS2*) suggesting that its anti-inflammatory potential is not limited to suppression of IL-8 production. In addition to inhibiting stimulation by OxPAPC, AZ11645373 suppressed induction of IL-8 by TNFα and LPS. To summarize, AZ11645373 inhibits in a P2X7-independent manner action of chemically different inflammatory agonists such as OxPLs, LPS, and TNFα. Thus, AZ11645373 may be especially effective for treatment of inflammatory disorders due to a beneficial combination of P2X7 receptor-dependent effects (inhibition of inflammasome activation, antinociceptive effects) with P2X7-independent general anti-inflammatory action described in this paper.

## INTRODUCTION

P2X7 receptor antagonists demonstrate antinociceptive and anti-inflammatory action and some of them are now in clinical trials for treatment of chronic inflammatory diseases [1–3]. It is generally accepted that the anti-inflammatory action of P2X7 receptor antagonists is mediated through the inhibition of P2X7 receptor-dependent activation of the NLRP-3 inflammasome [4–6]. Activation of P2X7 receptors by extracellular adenosine triphosphate induces efflux of potassium ions from cells resulting in activation of caspase-1, which processes IL-1β and IL-18 precursor proteins to the mature active cytokines [7]. In this work, we have tested a known P2X7 receptor antagonist AZ11645373 for its ability to inhibit induction of an inflammatory cytokine IL-8 in human endothelial cells. Here, we show that AZ11645373 inhibits induction of IL-8 by oxidized phospholipids, LPS, or TNFα and that this inhibitory effect is an off-target phenomenon independent of its action on P2X7 receptor.

## MATERIALS AND METHODS

### Preparation of OxPAPC

1-Palmitoyl-2-arachidonoyl-*sn*-glycero-3-phosphocholine (Avanti Polar Lipids; Alabaster, AL, USA) was oxidized by exposure to air for 24 h until approx. 20% of the phospholipid remained intact. The oxidation progress was monitored by thin-layer chromatography and by mass spectrometry [8].

### Cell Culture

Human umbilical vein endothelial cells (HUVECtert) were cultured in Nunclon^TM^ Delta surface flasks (Bartelt; Graz, Austria) in medium 199 (Gibco; Carlsbad, CA, USA) supplemented with 20% fetal calf serum (FCS) (Sigma-Aldrich; St. Louis, MO, USA), penicillin-streptomycin-amphotericin B (Lonza; Basel, Switzerland), and ECGS-heparin (PromoCell; Heidelberg, Germany) in the 95% humidified atmosphere with 5% CO_2_ at 37 °C. Stimulation of cells was performed in endothelial basal medium-2 (EBM-2) (Lonza) supplemented with 2% FCS (Sigma-Aldrich), and 20 mM HEPES (Lonza). The cells were preincubated with an inhibitor solution for 20 min. Then, an agonist solution was added to the cells. After 7 h of the incubation at 37 °C, cell culture supernatants were collected for ELISA. At the end of the experiment, viability of the remaining cells was measured using a tetrazolium salt (XTT) assay. Following inhibitors and agonists used were purchased from Sigma-Aldrich: AZ11645373, A740003, Ac-YVAD-cmk, LPS 005:B5, ATP, BzATP. Recombinant human TNFα was from PeproTech (Rocky Hill, NJ, USA).

### IL-8 ELISA

IL-8 in cell culture supernatants after the stimulation of cells was detected by a CXCL8/IL-8 DuoSet ELISA Kit (R&D Systems; Minneapolis, MN, USA) according to manufacturer’s instruction. Briefly, MaxiSorp 96-well plates (ThermoScientific; Waltham, MA, USA) were pre-coated with the capture antibody (4 μg/ml in PBS), incubated for 2 h at rt, and washed four times with 0.05% Tween 20 (Sigma-Aldrich) in PBS (*v*/*v*) using a HydroSpeed^TM^ washer (Tecan Group Ltd; Männedorf, Switzerland). Blocking was performed with 1% bovine serum albumin (BSA) (Carl Roth; Karlsruhe, Germany) in PBS (*w*/*v*) for 1 h at rt. After washing, supernatants or IL-8 standard were added to wells and incubated for 2 h at rt followed by another washing step. The detection antibody was diluted to 20 ng/ml with 0.1% BSA/0.05% Tween 20 in PBS (*w*/*v*/*v*), added to the plate and incubated for 1 h at rt. After another washing step, streptavidin-HRP was added and incubated for 30 min at rt, followed by a final washing step. The substrate OPD (ThermoScientific) was added and incubated at 37 °C in the dark until the color development. Absorbance was periodically measured using a multimode plate reader EnSight^TM^ (Perkin Elmer; Waltham, MA, USA) at 450 nm to avoid values above 0.4. After addition of 1 M sulfuric acid (ThermoScientific) to stop the reaction, final absorbance was measured at 492 nm.

### Cell Viability Assay—XTT

At the end of cell stimulation, metabolic activity of cells was determined. Triton X-100 (0.2% final concentration) was added to cells for 15 min for the negative control. Medium was exchanged to medium 199 with 0.2 mg/ml 2,3-bis-(2-methoxy-4-nitro-5-sulfophenyl)-2H-tetrazolium-5-carboxanilide (Molecular Probes; Eugene, OR, USA) and 5 nM phenazine methosulfate (Acros-Organic; Geel, Belgium). After 4 h of incubation at 37 °C, absorbance at 450 nm was measured.

### RNA Isolation and RT-qPCR

Cells were preincubated with a solution of AZ11645373 in EBM-2/2% FCS/20 mM HEPES for 20 min followed by addition of OxPAPC (30 to 40 μg/ml) in the same medium. After incubation for 6 h at 37 °C, the medium was removed and RNAzol®RT (Molecular Research Center Inc.; Cincinnati, OH, USA) was added and total RNA was isolated. Concentrations of RNA were determined using a DS-11+Spectophotometer (DeNovix; Wilmington, DE, USA).

Nine hundred nanograms of RNA were reverse-transcribed using murine leukemia virus reverse transcriptase (MuLV) and oligo(dT)_16_ primers (both from Applied Biosystems; Foster City, CA, USA). Complementary DNA (cDNA) was analyzed using a SYBRGreen master mix (PCR Biosystems Ltd; London, UK) on a StepOnePlus instrument (Applied Biosystems) using a comparative ΔC_t_ method and the amplification program as follows: 30 s at 90 °C, 40× (3 s at 95 °C, 30 s at 60 °C); melting point analysis in 0.1 °C steps. Sequences of primers used are β2-microglobulin (B2M, β2M) (fwd: 5′-ATT CAC CCC CAC TGA GAC TG-3′, rev: 5′-TGC TAT TTC TTT CTG CGT GC-3′), *IL-8* (fwd: 5′-CTC TTG GCA GCC TTC CTG ATT-3′, rev: 5′-TAT GCA CTG ACA TCT AAG TTC TTT AGC A-3′), *PTGS2* (fwd: 5′-CCG CAA ACG CTT TAT GCT GAA-3′, rev: 5′-TGG CCG AGG CTT TTC TAC CA-3′). Quantification of target gene expression was performed using a mathematical model by Pfaffl [9]. The expression of messenger RNA (mRNA) of target genes was normalized to the expression of β_2_-microglobulin mRNA. In all experiments, β_2_-microglobulin-normalized level of expression in control cells was taken as 1.

### Statistical Analyses

The results are representative of two to four independent experiments. Mean values and standard deviations for at least three parallels are presented. A one-way ANOVA analysis with the Tukey post hoc correction was used for calculation of statistical significance. Values with *p* ≤ 0.05 were considered as statistically significant.

## RESULTS

**Fig. 1:**
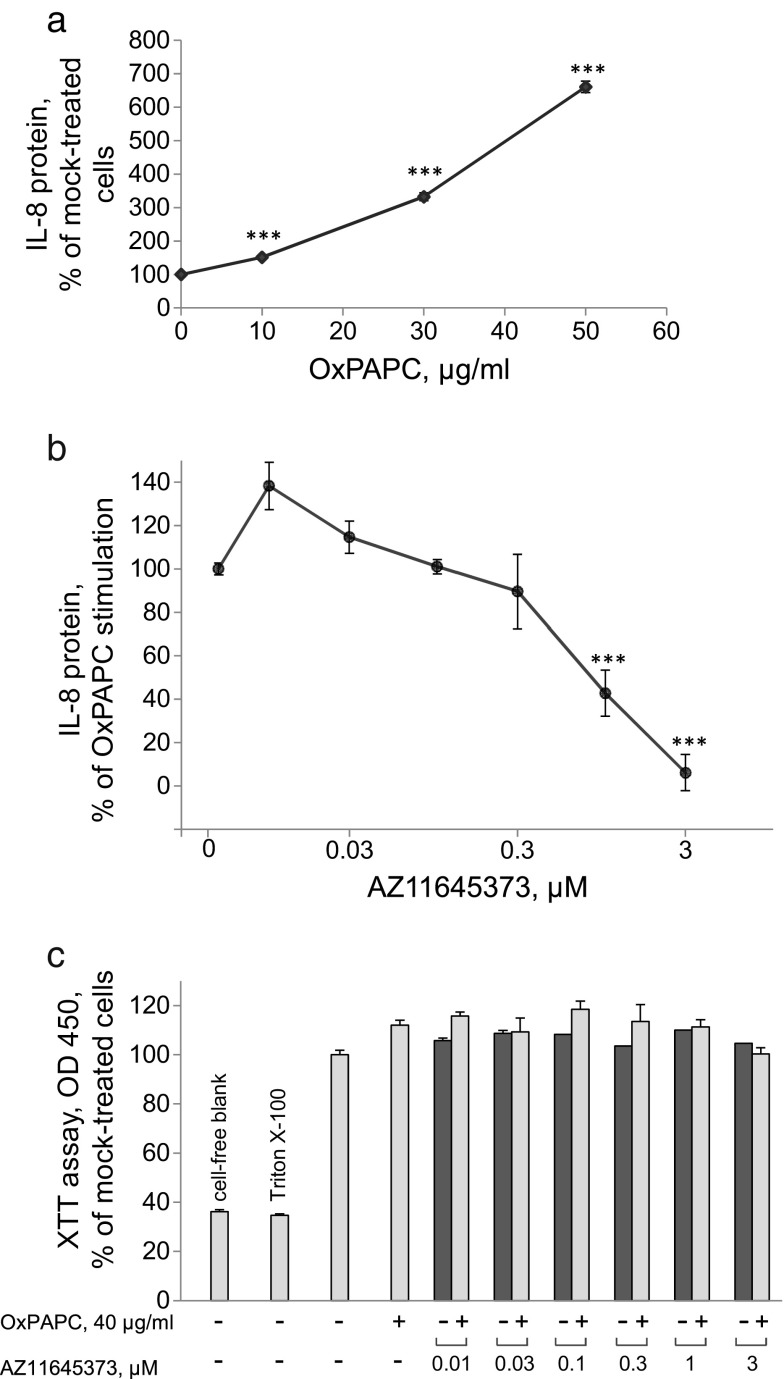
AZ11645373 inhibits OxPAPC-induced IL-8 release in endothelial cells. **a** OxPAPC induces IL-8 release. HUVECtert cells were stimulated by OxPAPC at the indicated concentrations for 6 h in EBM-2/2% FCS/20 mM HEPES in a 96-well plate. The concentrations of IL-8 released into the culture medium were measured by ELISA. IL-8 produced by mock-treated cells (26 ± 1.1 pg/ml) was taken as 100%. ***p < 0.005 (treatment vs mock). **b** AZ11645373 inhibits OxPAPC-stimulated upregulation of IL-8. After preincubation of HUVECtert with different concentrations of AZ11645373 in the same medium as above for 20 min, OxPAPC (40 μg/ml final concentration) was added. After 7 h, the cell culture supernatants were collected and the IL-8 concentrations determined by ELISA. IL-8 released after stimulation of cells with OxPAPC (155 ± 3.2 pg/ml) after subtraction of mock-treated control (40 ± 1.0 pg/ml) is expressed as 100%. ***p < 0.005 (vs OxPAPC without inhibitor). The remaining cells were analyzed by the XTT assay (**c**). A solution of XTT/PMS was added to cells and incubated for another 2 h at 37 °C followed by absorbance measurement at 450 nm. Cells pretreated with 0.2% Triton X-100 for 15 min before the addition of the XTT/PMS solution represent metabolically inactive cells. Blank values were measured in cell-free wells. The results are expressed as percentage of mock-treated cells (no OxPAPC, no inhibitor).

**Fig. 2:**
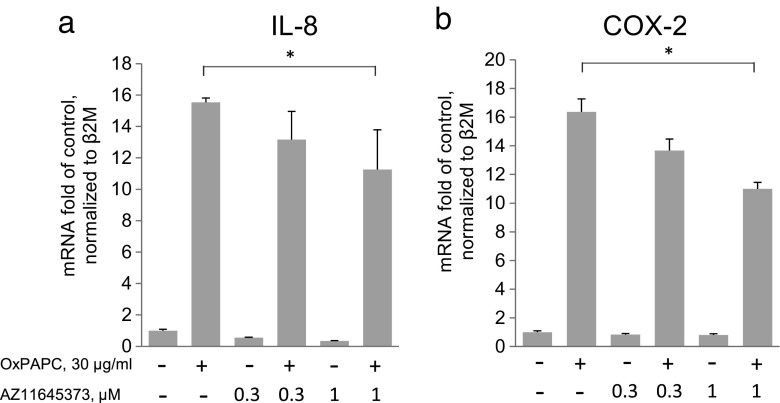
AZ11645373 inhibits OxPAPC-induced upregulation of IL-8 (**a**) and COX-2 (**b**) mRNA in endothelial cells. HUVECtert cells were preincubated with AZ11645373 in EBM-2 medium containing 2% FCS and 20 mM HEPES at 37 °C. After 20 min, OxPAPC was added and the cells were further incubated for 7 h. The isolation of total RNA, cDNA synthesis, and real-time PCR were performed as described in the “Materials and Methods” section. Results are normalized to β2-microglobulin. *p < 0.05.

Oxidized phospholipids such as oxidized 1-palmitoyl-2-arachidonoy-*sn*-3-glycerophosphocholine (OxPAPC) accumulate within the atherosclerotic lesion and induce pro-inflammatory activation of endothelial cells [10] including upregulation of chemokine IL-8 [11]. Concentration-dependent upregulation of IL-8 by OxPAPC in HUVECtert cells is illustrated in Fig. 1a. We found that the IL-8 release was blocked by an antagonist of P2X7 receptor, AZ11645373 (Fig. 1b). The inhibitory effect was not due to the toxicity of AZ11645373 as documented by the metabolic activity (XTT) assay performed at the end of the experiment (Fig. 1c). In addition to the downregulation of IL-8 protein, also IL-8 mRNA levels were decreased (Fig. 2a). OxPAPC-induced upregulation of another pro-inflammatory gene, COX-2 (*PTGS2*) was also suppressed by AZ11645373 (Fig. 2b). The data allow hypothesizing that AZ11645373 inhibited general pro-inflammatory mechanisms that are upstream of IL-8 and COX-2 (*PTGS2*) gene transcription.

**Fig. 3:**
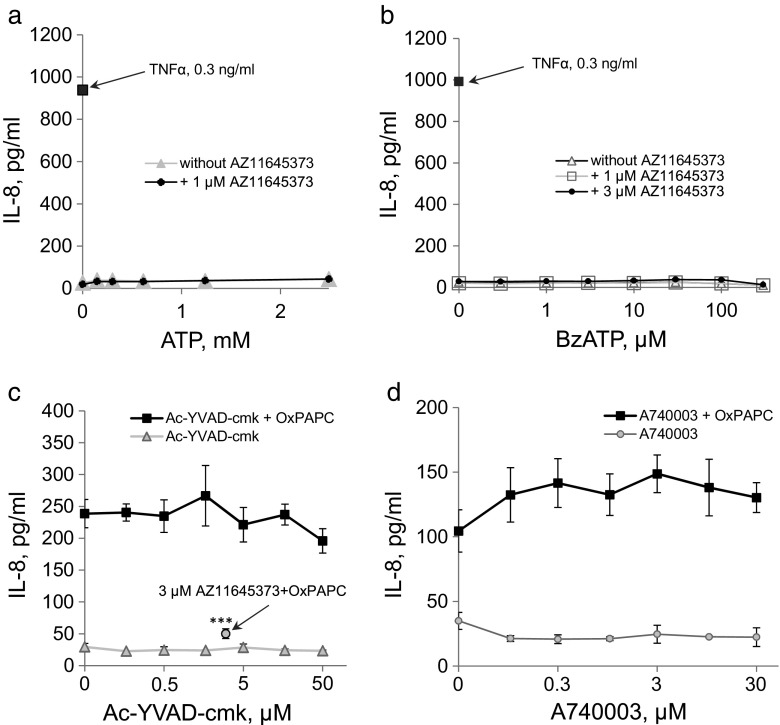
P2X7 receptor is not involved in the IL-8 release by ECs. HUVECtert were preincubated with AZ11645373 (final concentration 1 or 3﻿ μM) for 20 min at 37 °C followed by addition of ATP (**a**) or BzATP (**b**). After 7 h, the cell culture supernatants were collected and analyzed by the IL-8 ELISA. **c** Upregulation of IL-8 by OxPAPC is not dependent on caspase-1. HUVECtert were pretreated with a caspase-1 inhibitor (Ac-YVAD-cmk, final concentrations are indicated) or AZ11645373 (3 μM) for 20 min, followed by addition of OxPAPC (40 μg/ml). After 7 h, the cell culture medium was collected and analyzed by IL-8 ELISA. ***p < 0.005 (vs OxPAPC alone). **d** HUVECtert were preincubated with A740003 (final concentrations are indicated) for 20 min. After addition of OxPAPC (40 μg/ml), cells were incubated for further 7 h. The collected supernatants were analyzed by IL-8 ELISA.

AZ11645373 was described as a high-affinity antagonist of human P2X7 receptor [12]. In order to test if indeed the inhibitory action of AZ11645373 was mediated by its antagonism of the P2X7 receptor, we used two approaches. First, we checked if the P2X7 receptor is involved in upregulation of IL-8. The data of Fig. 3a, b demonstrate that two P2X7 receptor agonists, namely ATP and its higher-affinity-derivative BzATP, did not significantly upregulate IL-8. The data suggest that under the conditions of our experiments the induction of IL-8 was independent of the P2X7 receptor. This hypothesis is in general agreement with the known involvement of P2X7 receptors in activation of NLRP3 inflammasome that produces two major cytokines—IL-1β and IL-18 through caspase-1-dependent mechanism [7] that is different from the mechanisms inducing IL-8 expression. In agreement with these arguments, a caspase-1 inhibitor Ac-YVAD-cmk did not inhibit induction of IL-8 by OxPAPC (Fig. 3c). As a second approach for testing the role of P2X7 receptor in IL-8 induction, we applied a chemically different P2X7 agonist A740003. As illustrated by Fig. 3d, A740003 did not inhibit IL-8 release induced by OxPAPC thus suggesting that the inhibitory action of AZ11645373 on IL-8 induction was an off-target effect independent of P2X7 receptor.

**Fig. 4:**
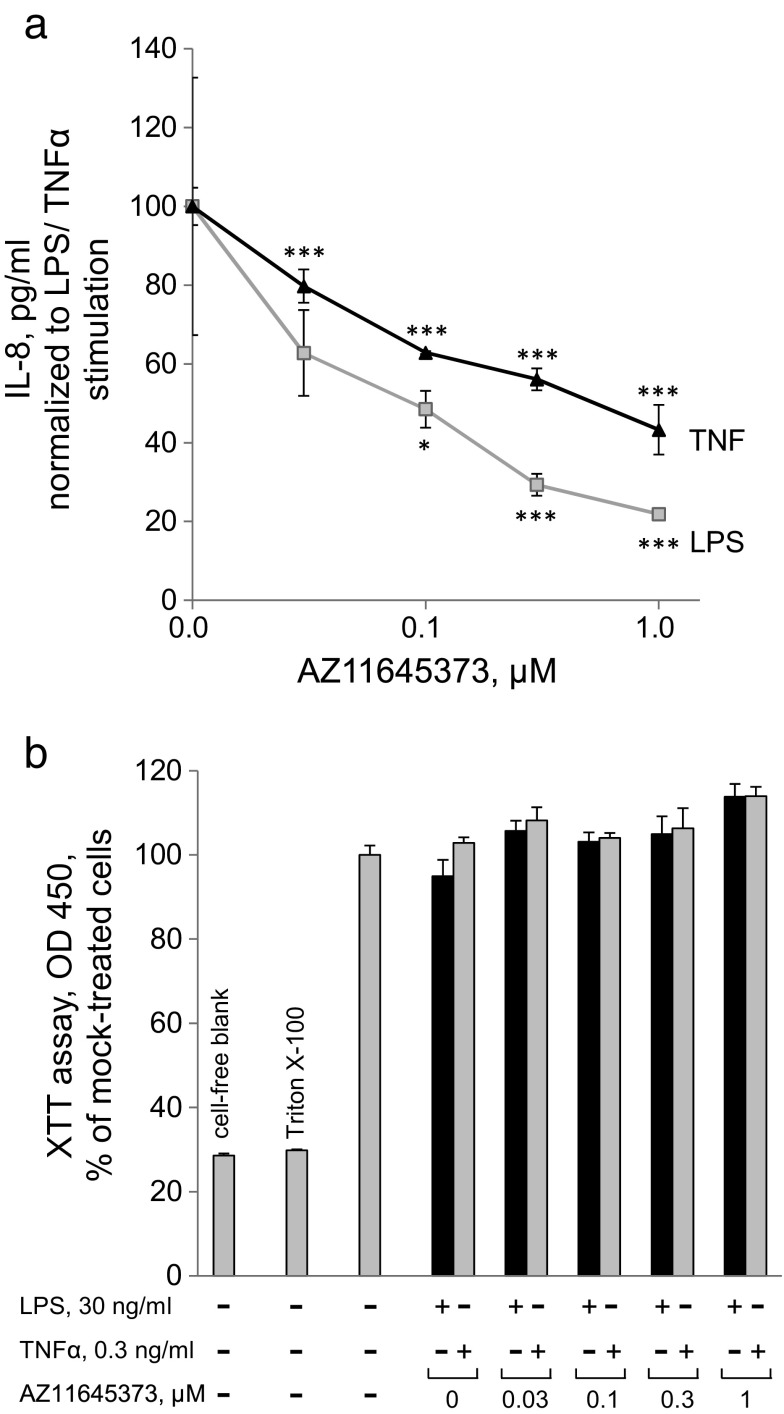
AZ11645373 prevents LPS- and TNFα-induced IL-8 release in endothelial cells. HUVECtert were pretreated with AZ11645373 at different concentrations for 20 min followed by addition of LPS (30 ng/ml) or TNFα (0.3 ng/ml). After 7 h, the cell culture supernatants were collected and analyzed by IL-8 ELISA (**a**). IL-8 release in the absence of inhibitor was 177 ± 46.4 pg/ml (LPS), 910 ± 45.2 pg/ml (TNFα) and 49 ± 4.4 pg/ml (mock-treatment). The respective IL-8 release by LPS or TNFα was set as 100%. *p < 0.05, ***p < 0.005 (vs LPS/TNFα treatment alone) (**b**). The cells remaining after the medium collection were treated with a XTT/PMS solution for analysis of metabolic activity as described in the legend to Fig. 1c.

We next addressed a question whether AZ11645373 can inhibit action of other pro-inflammatory stimuli like LPS and TNFα. Both LPS and TNFα stimulated IL-8 secretion, which was inhibited by AZ11645373 (Fig. 4a). The inhibitory effect did not result from the toxic action of AZ11645373 because the drug did not impair metabolic activity of cells determined by a XTT assay at the end of the experiment (Fig. 4b).

## DISCUSSION

A novel finding done in this work is that a P2X7 antagonist AZ11645373 inhibited expression of inflammatory molecules induced by chemically different classes of agonists including inflammatory cytokines, bacterial PAMPs (lipopolysaccharide), and endogenously generated DAMPs (lipid oxidation products such as OxPAPC). In addition to IL-8, AZ11645373 inhibited induction of COX-2 (*PTGS2*) gene and thus has a potential to block multiple facets in initiation and propagation of inflammation.

The mechanism of the broad anti-inflammatory action of AZ11645373 is not known. Sathanoori *et al.* have recently shown that pharmacological inhibition or knockdown of P2X7 and P2X4 receptors prevented upregulation of inflammatory cytokines in cells cultured for 48 h in the presence of high concentrations of glucose and palmitic acid [13]. The authors provided mechanistic evidence showing that glucose/palmitic acid treatment increased concentrations of extracellular ATP thus leading to the activation of P2X7-dependent pro-inflammatory pathways. Our data however point to the existence of an alternative mechanism of anti-inflammatory action of AZ11645373, which is P2X7 receptor-independent. Indeed, under our experimental conditions P2X7 receptor agonists ATP or BzATP did not induce IL-8. Furthermore, another chemically different P2X7 antagonist (A740003) did not inhibit elevation of IL-8 induced by OxPAPC. The data suggest that AZ11645373 inhibits inflammation independently of P2X7 receptor, *i.e*., through an off-target mechanism. The inhibition was observed at submicromolar concentrations of AZ11645373, which makes non-specific physicochemical mechanisms unlikely. One plausible explanation is that AZ11645373 binds to an alternative intracellular target different from P2X7 receptor and thus inhibits pro-inflammatory signaling pathways. The major pro-inflammatory transcription factor NF-κB is unlikely to be the (only) target of AZ11645373. Although the NF-κB-driven transcription plays a central role in the induction of IL-8 by TNFα and LPS [14] OxPAPC does not activate NF-κB [11, 15] and induces IL-8 through multiple signaling and transcriptional mechanisms including c-Src, STAT3, and ATF4 [16–22]. At the moment, there are no indications that any of these proteins can be inhibited by AZ11645373. Therefore, further pharmacological and target fishing studies are required in order to identify protein targets of AZ11645373 mediating its P2X7-independent anti-inflammatory action.

In summary, our data suggest that AZ11645373 in addition to its well-characterized ability to inhibit pro-inflammatory action of ATP demonstrates a broad P2X7 receptor-independent anti-inflammatory activity against chemically different types of inflammatory agonists. This type of polypharmacology may be especially effective for treatment of inflammatory disorders due to a combination of P2X7-dependent and P2X7-independent anti-inflammatory mechanisms. In other words, AZ11645373 has a potential to induce several beneficial effects including inhibition of inflammasome-mediated generation of IL-1β and IL-18, inhibition of inflammatory pain, as well as broad anti-inflammatory action described in this work.

BSA, bovine serum albumin; DAMP, danger-associated molecular pattern; EBM, endothelial basal medium; FCS, fetal calf serum; HUVEC, human umbilical vein endothelial cell; IL-8, interleukin-8; LPS, lipopolysaccharide; OxPL, oxidized phospholipid; PAMP, pathogen-associated molecular pattern; PAPC, 1-palmitoyl-2-arachidonoyl-*sn*-glycero-3-phosphocholine; TNF, tumor necrosis factor.
